# Editorial: Optimizing photosynthetic efficiency for sustainable crop production in varied climatic conditions

**DOI:** 10.3389/fpls.2025.1765839

**Published:** 2026-01-07

**Authors:** Ramwant Gupta, Bibi Rafeiza Khan

**Affiliations:** 1 Biophysics and Photosynthesis Laboratory, Department of Botany, Deen Dayal Upadhyaya (DDU) Gorakhpur University, Gorakhpur, India; 2 Biology Department, University of Scranton, Scranton, PA, United States

**Keywords:** climate change, crop productivity, food security, photosynthetic efficiency, photosynthetically active radiation

Photosynthesis continues to set the ultimate limit on crop productivity, and the ability of plants to maintain efficient light use, electron transport, and carbon assimilation under variable environments remains central to global food security. As climate change intensifies environmental fluctuations in temperature, light, and water availability, crops are increasingly challenged to sustain growth and yield under conditions that strain their photosynthetic capacity. This Research Topic was developed to highlight current advances in understanding how photosynthetic processes adjust and contribute to crop resilience across a wide range of climatic conditions.

This Research Topic brings together four studies that approach this challenge from complementary perspectives. Together, they show that photosynthetic performance cannot be defined by a single trait but emerges from a coordinated system that integrates physiology, agronomy, and genetics. They also demonstrate how adjustments in crop management and experimental design can reveal new opportunities to enhance photosynthetic resilience and productivity.

The first article, *Effects of Soybean Intercropping Density on Photosynthetic Characteristics and Disease Resistance in Tobacco*, investigates how soybean planting density influences photosynthetic function and disease resistance in tobacco. The authors show that a moderate soybean density improves the photosynthetic rate and PSII efficiency of tobacco, strengthens nitrogen metabolism, and reduces black shank disease incidence, resulting in higher quality and economic return.

The second study, *Nitrogen Fertilization Rates Affect Quality and Curing Characteristics of Tobacco During the Harvesting Period Under Field Chilling Stress*, examines how nitrogen supply shapes leaf quality and curing behavior during chilling stress. Increasing nitrogen above the conventional rate improves pigment stability, antioxidant activity, moisture regulation, and the sensory attributes of cured leaves, offering a practical strategy to mitigate chilling-induced declines in leaf quality.

The third contribution, *Optimal Row Configuration in Jujube–Cotton Intercropping Systems Increases Cotton Yield by Enhancing Growth Characteristics and Photosynthetically Active Radiation in an Arid Region*, evaluates how different cotton row arrangements influence canopy structure and productivity. Increasing the number of cotton rows improves leaf area development, enhances light interception, and substantially raises cotton yield, while the four-row configuration provides the most balanced and productive system for both crops.

The final article, *Nighttime Fluorescence Phenotyping Reduces Environmental Variability for Photosynthetic Traits and Enables the Identification of Candidate Loci in Maize*, presents an innovative phenotyping approach using nighttime chlorophyll fluorescence measurements. By reducing environmental noise through natural dark adaptation, this method increases the heritability of key fluorescence traits and improves the detection of genetic loci associated with photosystem efficiency.

Taken together, the studies in this Research Topic broaden our understanding of how photosynthesis responds to diverse environmental constraints and how crop management strategies can support more effective use of light and carbon resources. The Research Topic shows that progress emerges from approaches that connect molecular and physiological insights with field-level practices and genetic diversity.

Looking ahead, closer integration of field physiology with controlled environment analysis and modern phenotyping technologies will help build stronger links between mechanistic understanding and real-world crop performance. Greater attention to stomatal dynamics, canopy light distribution, chloroplast resilience, and the timing of senescence may also reveal new opportunities to strengthen photosynthetic capacity under stress. These perspectives demonstrate how photosynthesis research is expanding beyond the leaf to consider the broader agronomic and genetic context that ultimately determines yield.

We thank all authors and reviewers who contributed their work and expertise to this Research Topic. The papers presented here offer meaningful insights into the regulation of photosynthesis under variable climates and support the development of cropping systems capable of sustaining productivity in an increasingly unpredictable world.

